# Use of microbiome analysis as a complementary endpoint in clinical trials

**DOI:** 10.1016/j.isci.2025.113754

**Published:** 2025-10-14

**Authors:** Lourdes Velo-Suarez, Charles-Antoine Guilloux, Rozenn Le Berre, Stéphanie Gouriou, Gilles Rault, Dominique Mottier, Laurent Meijer, Geneviève Héry-Arnaud

**Affiliations:** 1 Center for Biome Analysis and Microbiota (CBAM), CHU Brest, 29200 Brest, France; 2 University Brest, Inserm, EFS, UMR 1078, GGB, 29200 Brest, France; 3 Département de Médecine Interne et Pneumologie, CHU Brest, 29200 Brest, France; 4 Fondation Ildys, Centre de Perharidy, 29684 Roscoff Cedex, France; 5 Centre d’Investigation Clinique (CIC 1412), CHU Brest, 29200 Brest, France; 6 ManRos Therapeutics, Presqu’île de Perharidy, 29680 Roscoff, France; 7 Unité de Bactériologie, Département des Agents Infectieux, CHU Brest, 29200 Brest, France

**Keywords:** Health sciences, Medical microbiology, Medicine, Respiratory medicine

## Abstract

The ROSCO-CF study evaluated the safety and effect of oral R-roscovitine in people with cystic fibrosis chronically colonized by *Pseudomonas aeruginosa*. While no direct impact on the respiratory pathogen was detected, lung and gut microbiomes were analyzed to explore broader effects of the treatment. Sputum and fecal samples collected before and after treatment were examined using 16S rDNA sequencing. Despite overall stability in alpha diversity, dose-dependent shifts in beta diversity suggested subtle restructuring of microbial communities. Temporal analyses indicated emerging patterns in microbial coordination at higher doses. Specific taxa, such as *Tannerella* and *Granulicatella elegans*, showed increased abundance, while *Streptococcus* decreased with dose. These results suggest that R-roscovitine may influence microbial dynamics in a personalized and dose-dependent manner, supporting the inclusion of microbiome profiling as an exploratory endpoint in clinical trials.

## Highlights

• Lung microbiome analysis shows dose-dependent shifts with R-roscovitine in cystic fibrosis
• No detected effect on *P. aeruginosa* infection in the initial clinical trial
• Highlights microbiome outcomes in early-phase trials of novel therapies

## Graphical abstract

**Figure:**
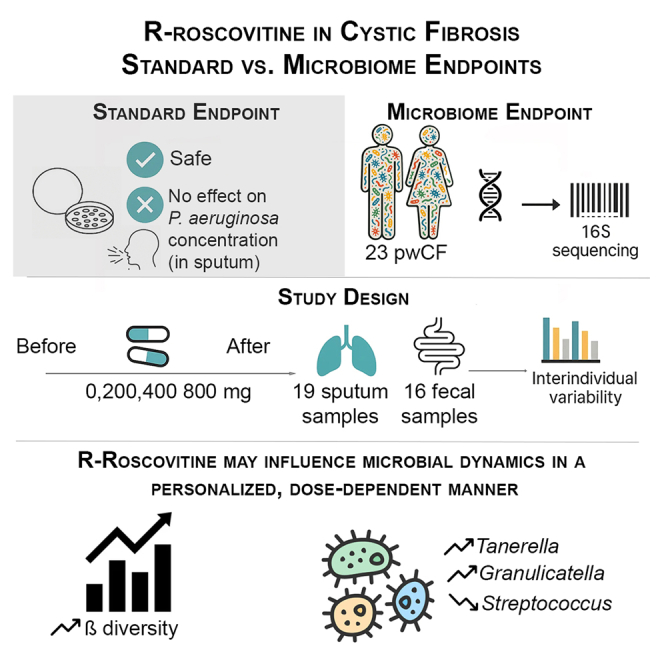

## Introduction

R-roscovitine (Seliciclib), a protein kinase inhibitor initially designed for cancer treatment,^1^^,^^2^ has been repurposed as a drug candidate in people with cystic fibrosis (pwCF)^3^ and tested in a study (NCT 01944735). ROSCO-CF was a multicenter, randomized, controlled, phase IIA, dose-ranging trial of R-roscovitine in 23 pwCF chronically infected with *Pseudomonas aeruginosa* (PA) with at least one F508del-CFTR mutation. The goal was to assess the safety of the drug and its potential beneficial effects. The European Medicines Agency (EMA) guidelines were used as conventional endpoints.^4^ R-roscovitine was found to be safe and rather well-tolerated by pwCF.^5^ Still, R-roscovitine showed no impact on PA infection based on EMA microbiological endpoints. The standard microbial endpoints of the EMA only consider monospecific absolute changes in PA. Given the potential mode of action of R-roscovitine, which, unlike antibiotics, does not directly target PA, it was valuable to verify the effect of this molecule on all bacteria of the lung and gut microbiomes.

## Results and discussion

### Gut stability and lung microbiome dose-related shifts with R-roscovitine

Substantial interindividual variability emerged as a defining feature of the microbiome profiles in this cohort, despite all participants being chronically colonized with PA at recruitment. NGS data revealed highly contrasting lung microbiomes, where PA did not always dominate (Figure S2). These results align with recent findings^6^ that demonstrate the need to stratify study groups (treatment versus placebo) according to their microbiome profile, even before the clinical trial begins. These differences in microbial lung communities may result in the heterogeneity in biological parameters measured in the patients included in the clinical trial.^5^ Consistent with this microbial heterogeneity, linear mixed-effects models showed notable interindividual variability across all indices.

Stool results indicate that placebo and R-roscovitine patients exhibited similar rates of change in alpha and beta diversities (Figure S3). These data suggest that R-roscovitine has no detectable short-term impact on gut microbiomes. This observation aligns with prior studies on CFTR modulators, which also report limited or delayed effects on gut microbial composition. For example, Pope et al. did not observe any significant gut microbiome changes after four months of ivacaftor treatment,^7^ while Kristensen et al. found increased alpha diversity only after at least one year of therapy.^8^ The short duration of R-roscovitine treatment in the current study (four weeks) is therefore likely insufficient to elicit measurable changes, particularly in the context of a relatively stable gutmicrobiota shaped by lifelong antibiotic exposure and the underlying CF defect. These factors may limit responsiveness to short-term interventions. While no immediate changes were observed, we cannot exclude the possibility that longer-term or cumulative effects of R-roscovitine on gut microbial communities may emerge over time. Notably, similar delayed responses have been reported for CFTR modulators in the lung microbiome as well.^8^^,^^9^^,^^10^^,^^11^^,^^12^

While global analyses on alpha diversity did not detect significant shifts, multiple complementary approaches consistently pointed toward dose-related microbial restructuring in the lung. Bray-Curtis dissimilarities (beta diversity) between pre- and post-treatment samples increased with R-roscovitine dose, with the highest values observed in the 800 mg group. A Kruskal-Wallis test showed a near-significant difference across treatment groups (χ^2^ = 7.712, *p* = 0.052). Pairwise Wilcoxon tests revealed low *p* values in comparisons involving the high-dose group (*p* = 0.095 vs. placebo, *p* = 0.071 vs. 200 mg, *p* = 0.065 vs. 400 mg), suggesting a dose-dependent trend in beta diversity (Figure S3B).

However, when using stratified PERMANOVA, which accounts for inter-individual variability, no significant differences were found across treatment groups (R^2^ = 0.12, F = 0.85, *p* = 0.89). Similarly, linear mixed-effects models fitted to the first three PCoA axes (derived from Bray-Curtis distances) did not identify significant fixed effects of dose or time. This suggests that global shifts in microbial composition were not consistent enough across individuals to be detected using community-level multivariate models.

To further explore individual-level dynamics, we applied the non-parametric microbial interdependence test (NMIT), which evaluates changes in the temporal coordination of microbial taxa within each subject. NMIT revealed a trend toward distinct microbial trajectories in the high-dose group (F = 1.18, R^2^ = 0.20, *p* = 0.061), supporting the hypothesis that increasing R-roscovitine doses may influence the internal structure and dynamics of the microbiome, even if overall diversity or composition remains statistically unchanged at the group level. Together, these analyses suggest that R-roscovitine may induce individualized and dose-related shifts in microbial community dynamics that are not fully captured by global metrics alone.

### Taxa-level shifts suggest potential beneficial modulation

While global diversity metrics and multivariate models failed to detect consistent shifts across individuals, the NMIT analysis revealed emerging patterns in temporal microbial dynamics, particularly in the high-dose group. These results suggest that R-roscovitine may not lead to uniform community-wide restructuring, but rather induces individualized changes in the coordination and behavior of specific microbial populations. To further investigate these targeted effects, we shifted from community-level analyses to feature-level modeling. We applied MaasLin2 to identify individual microbial taxa whose relative abundances were significantly associated with treatment dose, allowing us to detect finer-scale dose-responsive patterns that may underlie the subtle restructuring observed with NMIT.

**Figure 1:**
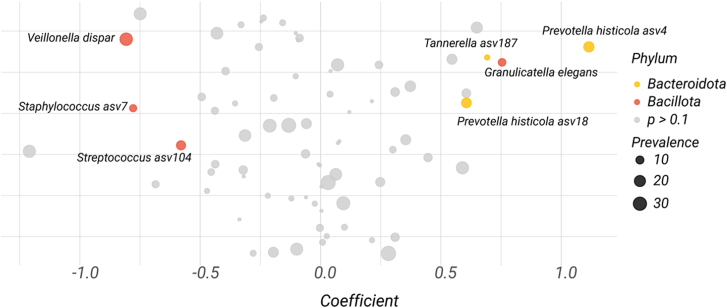
Differential abundance of microbial features associated with increasing dose of R-roscovitine, as identified by MaAsLin2 Each point represents a microbial amplicon sequence variant (ASV), plotted by its effect size (coefficient) on the *x* axis. Bubble size indicates prevalence (number of samples in which the feature was detected). ASVs with *p* < 0.1 are colored by bacterial phylum; non-significant features are shown in gray. Labels display the genus and species of significant features (or genus and ASV ID if species is unclassified). Positive coefficients indicate features whose abundance increases with dose (relative to placebo), while negative coefficients indicate features that decrease in abundance with increasing dose.

Notably, *Tannerella* (asv187) and *Granulicatella elegans* (asv65) showed positive associations with increasing dose (coefficients 0.69 and 0.75, respectively; *p* < 0.01), indicating that their relative abundances increased with higher treatment doses (Figure 1). Conversely, *Streptococcus* (asv104) exhibited a significant negative association with dose (coefficient −0.58, *p* = 0.02), suggesting a decrease in abundance as dose increased (Figure 1). These associations were detected across multiple taxa within the Bacteroidota and Bacillota phyla, specifically within orders Bacteroidales and Lactobacillales. Other features, such as *Prevotella histicola* (asv4 and asv18) and *Staphylococcus* (asv7), showed trends toward dose association but did not reach statistical significance after correction for multiple testing. Our findings are consistent with those of Bernarde et al.,^13^ who reported an increase in *Prevotella* abundance in the lungs following ivacaftor therapy, correlating this shift with improved pulmonary function. In addition, *Staphylococcus aureus* is one of four taxons corresponding to well-established CF pathogens (alongside PA, *Stenotrophomonas maltophilia*, and the *Burkholderia cepacia* complex) that have been implicated in the major pulmonary pathological states previously described in CF.^14^ The presence of *Tannerella* in the lung microbiome has been suggested to exert immunomodulatory effects, potentially contributing to the maintenance of respiratory homeostasis.^15^ Anaerobic genera such as *Prevotella* or *Granulicatella* are recurrently associated with stable clinical status and better lung function in pwCF.^16^^,^^17^ In this context, our findings suggest that R-roscovitine may exert a beneficial modulatory effect on the lung microbiome of pwCF, marked by an enrichment in several anaerobic commensals and a concomitant reduction in *Staphylococcus* abundance.

### Toward patient-stratified, microbiome-guided trial design

Beyond these microbial shifts, our study emphasizes the critical importance of assessing microbiome-related outcomes when evaluating novel therapeutic agents in early-phase clinical trials. Such analyses provide complementary insights that may escape detection through conventional EMEA clinical endpoints, thereby contributing to a more holistic understanding of treatment impact.

A major limitation of this study is the small sample size, with only 23 participants. While such numbers are typical for Phase 2a trials in rare diseases like CF, they inherently limit statistical power and the generalizability of findings, particularly when interpreting subgroup effects such as dose-dependent changes. The limited number of samples per participant (pre- and post-treatment only) also constrains the ability to capture the full spectrum of microbial dynamics over time. Although previous work has shown that a single sample per collection day can reliably represent microbiome diversity,^6^ larger and more longitudinally sampled cohorts will be essential in future trials to validate these findings and detect more robust, reproducible signals of treatment-related microbial modulation.

Our findings highlight dose-dependent modulation of the lung microbiome, as evidenced by differential abundance patterns and shifts in microbial interaction dynamics identified through NMIT, suggesting potential microbial biomarkers or mechanistic effects of treatment. Future studies incorporating absolute quantification methods, such as qPCR for total and species-specific bacterial load, will be important to validate and contextualize these compositional shifts. In addition, integrating functional approaches such as metagenomics, proteomics, or metabolomics will be essential to determine the biological significance of these taxonomic changes and their potential impact on lung health. However, the significantly higher cost and complexity of multi-omics approaches can limit their feasibility in early-phase clinical trials, particularly in rare diseases, and should be carefully considered in future study designs.

To conclude, this proof-of-concept study supports the relevance of implementing microbiome community analysis for clinical research trials, especially for drugs with different mechanisms of action, such as R-roscovitine. Microbiome analysis enables a refined assessment of drug effects by identifying cryptic, induced biological phenomena. Understanding the microbiome of pwCF prior to future clinical trials would be beneficial in the context of patient stratification, trial design, and endpoints in the era of precision medicine. However, further studies will be needed with more participants to confirm these findings and assess their broader applicability across diverse patient populations.

### Limitations of the study

This study involved a small number of participants, which limits statistical power and generalizability of the results. The analysis relied on only two time points per subject, preventing detailed assessment of longitudinal microbiome dynamics. High interindividual variability in lung microbiome composition also limited the detection of consistent dose-related effects at the group level. In the gut microbiome, the short treatment duration may have been insufficient to induce detectable changes. Lastly, the study used relative abundance data without functional or absolute quantification, restricting interpretation of biological significance.

## Resource availability

### Lead contact

Further information and requests for resources and data should be directed to and will be fulfilled by the lead contact, Geneviève Héry-Arnaud.

### Materials availability

This study did not generate new unique reagents.

### Data and code availability

• Raw 16S rDNA sequencing data have been deposited in the NCBI sequence read archive under accession number PRJEB47611.
• Custom code used for bioinformatic and statistical analyses is available at: [https://github.com/lvelosuarez/Snakemake_amplicon] and [https://github.com/donaldtmcknight/microDecon].

## Acknowledgments

This study was supported by a “Programme Hospitalier de Recherche Clinique - National” (PHRC-National) (French Health Ministry, grant no PHRC-14-0117).

The authors would like to thank the study participants and families, and the French association Vaincre la Mucoviscidose. The authors would also like to thank the ROSCO-CF study group: Cyril Leven, Emmanuel Nowak, Sophie Hillion, Yves Renaudineau, Isabelle Durieu, Raphaël Chiron, Anne Prevotat, Isabelle Fajac, Dominique Hubert, Marlène Murris-Espin, Sandrine Huge, Isabelle Danner-Boucher, Bruno Ravoninjatovo, Sylvie Leroy, Julie Macey, Thierry Urban.

## Author contributions

Software, formal analysis, visualization, writing – original: L.V.-S.; investigation, writing – review and editing: C.-A.G.; methodology: R.L.B.; investigation: S.G.; methodology; resources, funding acquisition: G.R.; methodology: D.M.; methodology: L.M.; methodology, validation, writing – review and editing, resources, supervision: G.H.-A.

## Declaration of interests

Funding sources had no involvement in study design, collecting samples, analysis, and interpretation of data, or in writing or submission of the article. Laurent Meijer is the founder of ManRos Therapeutics and co-inventor of the “R-roscovitine and CF” patent.

## STAR★Methods

### Key resources table

**Table:** 

REAGENT or RESOURCE	SOURCE	IDENTIFIER
**Biological samples**		

Human sputum and stool samples	ROSCO-CF study	ClinicalTrials.gov: NCT02649751
Mock microbial community	Ozyme, France	Cat# ZD6310

**Chemicals, peptides, and recombinant proteins**		

R-roscovitine (Seliciclib)	Provided for clinical use	See protocol

**Critical commercial assays**		

DNA extraction kits	QIAamp DNA Fast Stool Mini Kit QIAamp DNA Mini Kit	Cat# 51604 Cat# 51306
Library preparation kit	MiSeq Reagent Kit v3 (600-cycle), Illumina	Cat# MS-102-3003

**Deposited data**		

Raw 16S rDNA sequences	NCBI SRA	PRJEB47611

**Software and algorithms**		

BBDuk (part of BBTools)	Bushnell B.	https://sourceforge.net/projects/bbmap/
DADA2	Callahan	https://benjjneb.github.io/dada2
dbOTU3	Preheim	https://github.com/kylebittinger/dbOTU3
SILVA v138 database	Quast et al.^18^	https://www.arb-silva.de
Snakemake_amplicon pipeline	Velo-Suarez	https://github.com/lvelosuarez/Snakemake_amplicon
microDecon	McKnight	https://github.com/donaldtmcknight/microDecon
NMIT (R package v1.0)	Zhao^19^	https://github.com/biobakery/NMIT
MaAsLin2	Mallick et al.^20^	https://github.com/biobakery/maaslin2
R software (v4.x)	R Foundation	https://www.r-project.org
Vegan, phyloseq, lme4 packages	R CRAN	https://cran.r-project.org

### Experimental model and subject details

#### Clinical cohort

The trial is registered on www.clinicaltrials.gov as NCT02649751, and has authorization from ANSM (French National Public Health and Drug Security Agency): 2015-002911-13/150937A-41.

The ROSCO-CF clinical study included pwCF receiving oral R-roscovitine. Nineteen patients contributed 38 sputum samples, and sixteen of them also provided 32 stool samples.

Samples were collected at two time points.

(1) T0 (Baseline): prior to first drug intake
(2) T1 (Post-treatment): after the 4-week treatment period

Treatment consisted of oral administration of R-roscovitine at three dosage levels: 200 mg, 400 mg, and 800 mg. A subsequent 4-week follow-up period was observed.

Comprehensive demographic data, including pancreatic sufficiency status, age, and gender, are detailed in ref.^5^. Temporal sample distribution and overlap between stool and sputum samples are illustrated in Figure S1.

### Method details

#### DNA extraction and sequencing

Microbial DNA from sputum and stool was subjected to 16S rDNA metagenomic profiling, targeting the V3-V4 region using Illumina MiSeq (2 × 300 bp, V3 chemistry). Quality control steps included.

(1) Filtering and trimming using BBDuk (Q ≥ 20; min length = 200 bp)
(2) Denoising with DADA2 pipeline
(3) OTU clustering via dbOTU3
(4) Taxonomic assignment using SILVA v138

Negative extraction controls and a commercial mock community (Ozyme, France) were included to monitor contamination and sequencing fidelity. Contaminants were filtered using the microDecon R package.

Post-processing, all samples were rarefied to 10,000 reads to account for differences in sequencing depth.

### Quantification and statistical analysis

#### Alpha diversity

Microbial richness and evenness were quantified using Chao1, Faith’s phylogenetic diversity, Shannon, and Simpson indices.

(1) Within-subject comparisons (T0 vs. T1) were analyzed using paired Wilcoxon signed-rank tests across dose groups.
(2) Linear mixed-effects models (LME) were applied with diversity metrics as outcomes, and dose, time, and their interaction as fixed effects. A random intercept for patient ID accounted for repeated measures.

#### Beta diversity

Bray–Curtis dissimilarities were computed to assess community composition changes.

A two-step approach was used.

(1) Kruskal–Wallis test for global differences across groups
(2) Pairwise Wilcoxon tests for specific contrasts

PERMANOVA with patient ID as a stratification variable tested for group-level effects of dose, time, and their interaction.

To visualize these patterns, PCoA was performed, and the first three axes were evaluated using LME models, including age as a covariate.

#### Longitudinal dynamics

The Non-parametric Microbial Interdependence Test (NMIT) was used to explore temporal trajectories of the lung microbiome. NMIT quantifies intra-individual interdependence patterns over time, allowing comparison of microbial network evolution across treatment arms.

#### Differential abundance

MaAsLin2 was used to identify microbial ASVs associated with R-roscovitine dose and treatment timepoint. The model included.

(1) Fixed effects: dose, time, and their interaction
(2) Random effect: patient ID

ASVs were retained if present in at least 10% of samples and with ≥0.0001 relative abundance.

*P*-values were adjusted for multiple testing to determine significance.
