# Supplementary material for: Use of microbiome analysis as a complementary endpoint in clinical trials
Source: iScience. 2025 Oct 14;28(11):113754. doi: 10.1016/j.isci.2025.113754 (PMC12637243; doi:10.1016/j.isci.2025.113754)
Supplement: Document S1. Figures S1–S3 [file mmc1.pdf]

iScience, Volume *28*

Supplemental information

Use of microbiome analysis

as a complementary endpoint in clinical trials

Lourdes Velo-Suarez, Charles-Antoine Guilloux, Rozenn Le Berre, Stéphanie Gouriou, Gilles Rault, Dominique Mottier, Laurent Meijer, and Geneviève Héry-Arnaud

Supplementary Materials

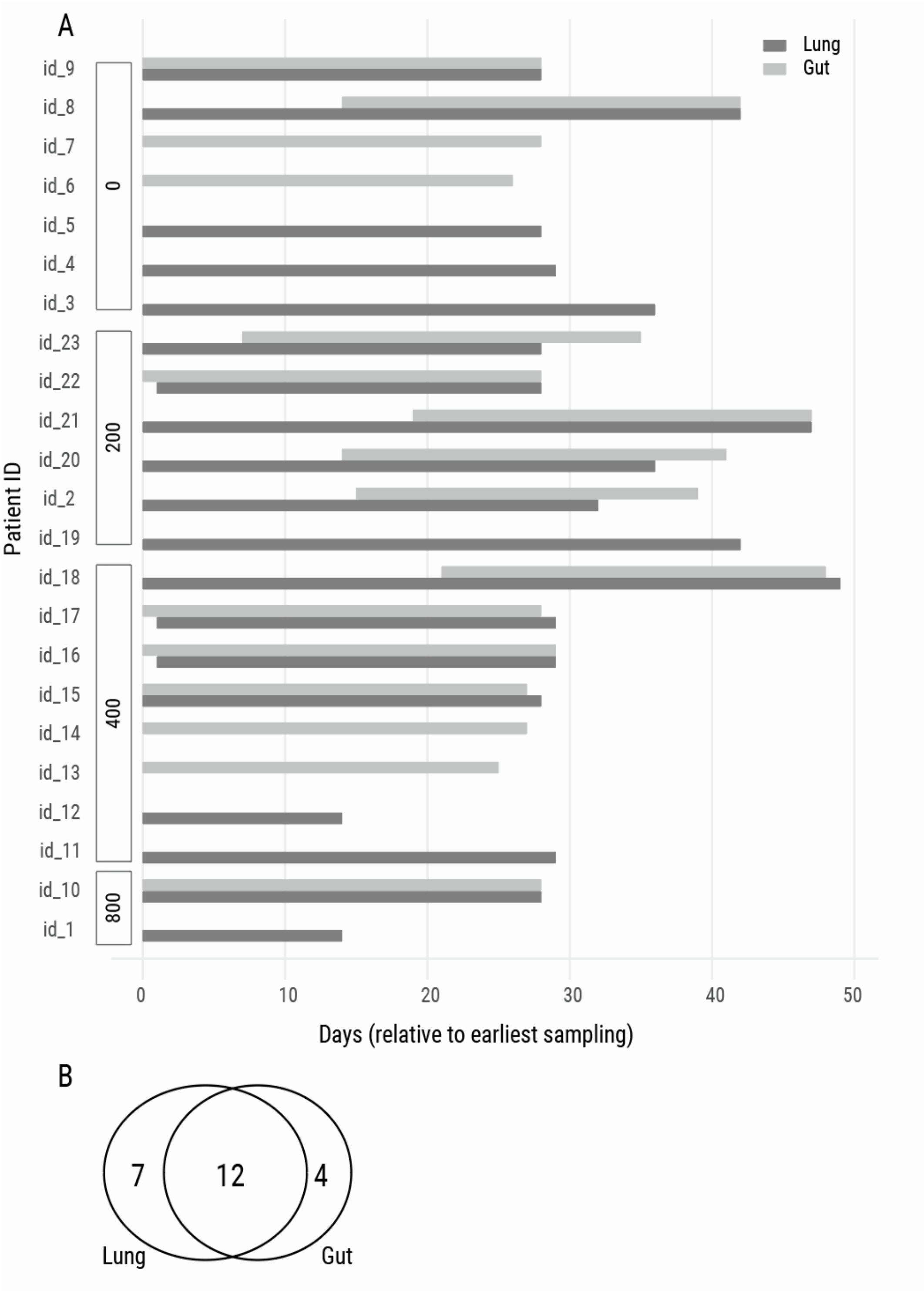

Figure S1. Sampling distribution and overlap of microbiome profiles between gut and lung.

A). Sampling timeline for each patient, stratified by treatment groups: 0, placebo, 200, 400, and 800 mg. Bars represent the duration of microbiome sampling for each patient, with gut samples indicated by light bars and lung samples by dark gray bars. Time is shown relative to the earliest sampling day (Day 0). B). Venn diagram showing the number of patients (n=23) with microbiome data available from lung only (n=7), gut only (n=4), and both lung and gut (n=12).

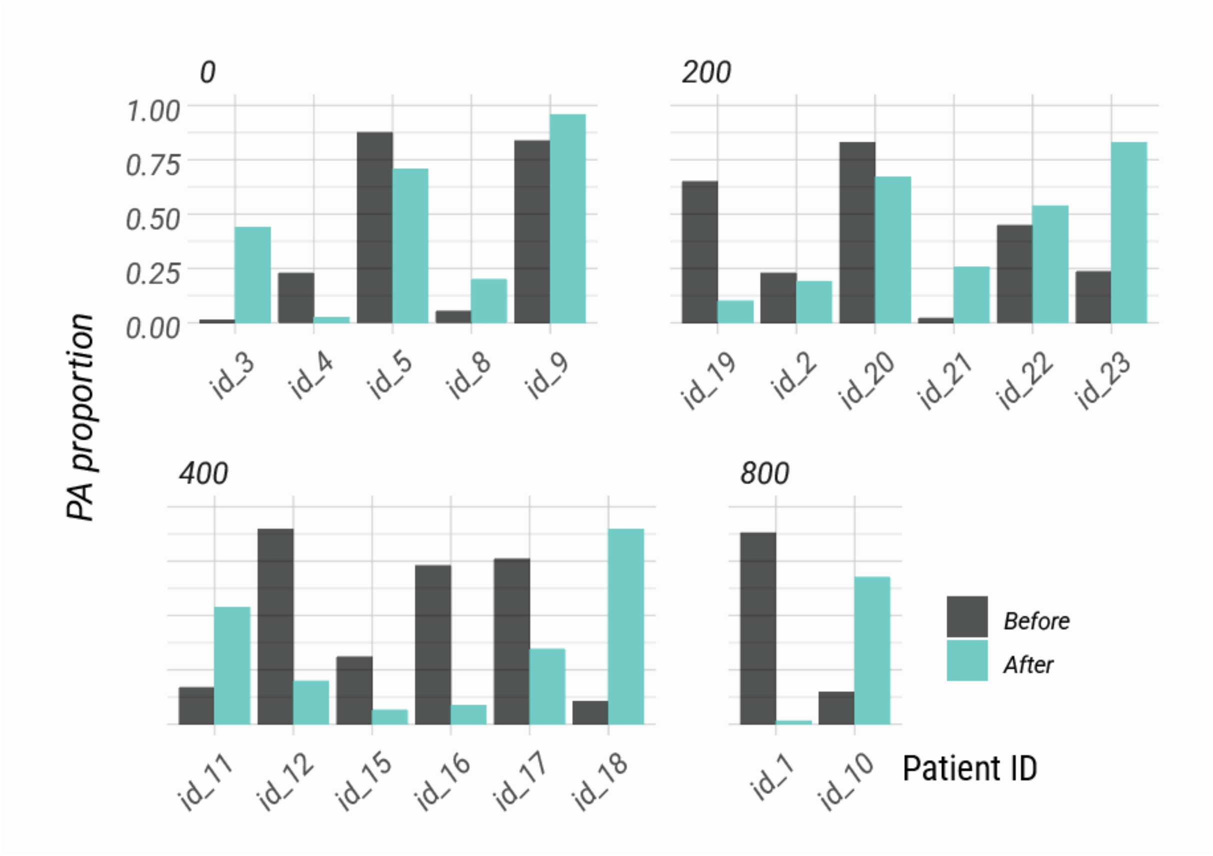

Figure S2. Proportion of *Pseudomonas aeruginosa (*PA) observed in patients before and after treatment with varying doses of R-roscovitine.
Panels represent data for different dose groups: 0 - placebo (top left), 200 mg (top right), 400 mg (bottom left), and 800 mg (bottom right). Individual anonymized patient IDs are shown on the x-axis. Bars represent measurements taken before (black) and after (blue) treatment.

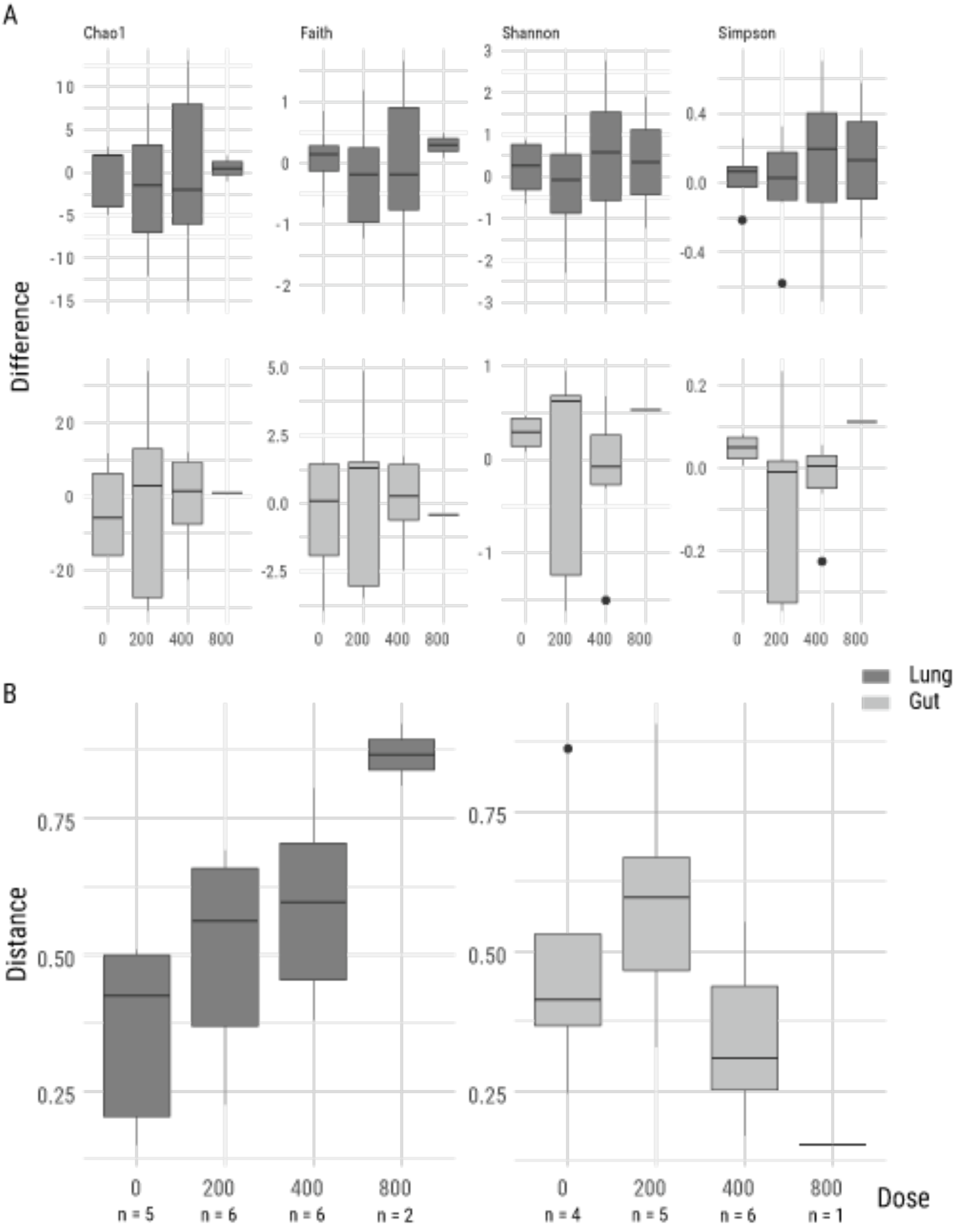

Figure S3. **Dose-dependent shifts in lung microbiome composition.** A). Pairwise comparison (before-after) of Chao1, Faith phylogenetic diversity, Shannon entropy, and Simpson diversity indexes of the lung (dark grey) and gut (light grey) microbiome. The X-axis shows differences for placebo (0) and roscovitine treatment (200, 400, and 800 mg) groups. B). Pairwise differences in Bray-Curtis distances. Sample pairs (from the same patient), before and after taking roscovitine: 0, placebo, 200, 400, and 800 mg for lung (dark grey) and gut (light grey). Boxplots show a quartile distribution of differences or distances between each subject's diversity. Note the significant increase in inter-sample variation between placebo and increased R-roscovitine doses.
